# Entrainment of mouse peripheral circadian clocks to <24 h feeding**/**fasting cycles under 24 h light/dark conditions

**DOI:** 10.1038/srep14207

**Published:** 2015-09-23

**Authors:** Yutaro Hamaguchi, Yu Tahara, Hiroaki Kuroda, Atsushi Haraguchi, Shigenobu Shibata

**Affiliations:** 1 Laboratory of Physiology and Pharmacology, School of Advanced Science and Engineering, Waseda University, Tokyo, Japan

## Abstract

The circadian clock system in peripheral tissues can endogenously oscillate and is entrained by the light-dark and fasting-feeding cycles in mammals. Although the system’s range of entrainment to light-dark cycles with a non-24 h (<24 h) interval has been studied, the range of entrainment to fasting-feeding cycles with shorter periods (<24 h) has not been investigated in peripheral molecular clocks. In the present study, we measured this range by monitoring the mouse peripheral PER2::LUCIFERASE rhythm *in vivo* at different periods under each feeding cycle (Tau (T) = 15–24 h) under normal light-dark conditions. Peripheral clocks could be entrained to the feeding cycle with T = 22–24 h, but not to that with T = 15–21 h. Under the feeding cycle with T = 15–18 h, the peripheral clocks oscillated at near the 24-h period, suggesting that they were entrained to the light-dark cycle. Thus, for the first time, we demonstrated the range of entrainment to the non-24 h feeding cycle, and that the circadian range (T = 22–24 h) of feeding stimulus is necessary for peripheral molecular clock entrainment under light-dark cycles.

The circadian clock system maintains the daily rhythm of physiological functions to contribute to the maintenance of homeostasis in mammals^1^. The suprachiasmatic nucleus (SCN) in the hypothalamus, the central pacemaker, orchestrates the local clocks in peripheral tissues (peripheral clocks)^2^. The rhythmic expression or functions of clock genes (e.g. *Per2*) play a role in the molecular mechanism underlying the ticking of the circadian clock at a single-cell level in each tissue. Light, food, and exercise are important external entraining cues to fix the time of internal clocks because the internal clocks can potentially tick time within a non-24-h period; however, this period must still be near to 24 h^3^. Time-restricted (scheduled) feeding paradigms have been used to understand the characteristics of food-induced entrainment in the circadian clock system^3^. Mice fed only at a specific time during the day exhibit food-seeking behaviour (food anticipatory activity) before the feeding time, which indicates that internal clocks respond to feeding times. Along with the behavioural change, almost all peripheral clocks alter their phases to the feeding time. However, the SCN clock does not shift because of the predominant light stimulus^4^,^5^.

In order to understand internal oscillator robustness and external entrainment cues, a T-cycle experiment was proposed. Altering the period of external entrainment cues forms a part of the T-cycle experiment. These experiments were performed for behavioural rhythms using light, running wheels, or food cues with non-24-h cycles (T-cycles) of entraining cues (periods either shorter or longer than 24 h). The range of entrainment of the T-cycle tells us the strength of the entrainment cue and the robustness of the oscillator. Large ranges of entrainment limits mean strong external entrainment cues and weak internal oscillators. The range of entrainment of light has been previously reported^6^,^7^, but this range differed among different species or experimental conditions (e.g. light intensity, with/without running wheels). However, the range was usually 24 ± 3 h for light-dark T-cycle experiments. A similar range of periods of adaptation to the T-cycle was observed for food anticipatory activity with scheduled feeding paradigms^8^,^9^,^10^,^11^. On the other hand, mice fed two (12-h interval) or three (8-h interval) meals a day at identical intervals exhibited food anticipatory activities before each meal in normal light-dark conditions^12^,^13^. This indicates that internal clocks can identify feeding times with 12- or 8-h cycles. Thus, it is interesting to elucidate whether a scheduled T-cycle feeding paradigm could entrain peripheral clocks with shorter T-cycle ranges (Tau (T) = 15–23 h). The SCN is not responsible for the scheduled feeding paradigm but plays a predominant role in the light-dark cycle^4^,^5^. Therefore, if the T-cycle of the feeding schedule could not entrain the peripheral clock, the phase of this clock may reflect the phase of the photic SCN-dependent rhythms because a normal light-dark cycle is maintained in the current experiment.

Previously, *in vitro* clock monitoring using tissue culture has been performed for luciferase reporter mice to study the effect of molecular clocks on the T-cycle of light. However, no correlation was noted between *in vivo* (behaviour) and *in vitro* (molecular clock) oscillations^14^,^15^. To address this discrepancy, we used a recently developed method for *in vivo* peripheral clock monitoring^16^.

Longer intervals of feeding cycles (T ≥ 24 h) mean longer fasting periods, and longer fasting periods followed by refeeding present a stronger stimulatory effect on the phase decision of the peripheral clocks^17^,^18^. As mentioned above, the anticipatory activity rhythm is entrained by shorter T-cycles like 12- or 8-h cycles. Considering such circumstances, in the present study, we monitored the phases and periods of peripheral clocks in various short T-cycles (T = 15, 18, 21, 22, 23, or 24 h) of scheduled feeding under standard light-dark conditions. The range of entrainment to the T-cycle (<24 h) of scheduled feeding in the peripheral molecular clocks had not been studied previously; thus, to the best of our knowledge, we are the first group to investigate this property.

## Results

### Experiment 1: Effects of scheduled feeding with T = 21–24-h cycles on the phase of peripheral clocks

**Figure 1:**
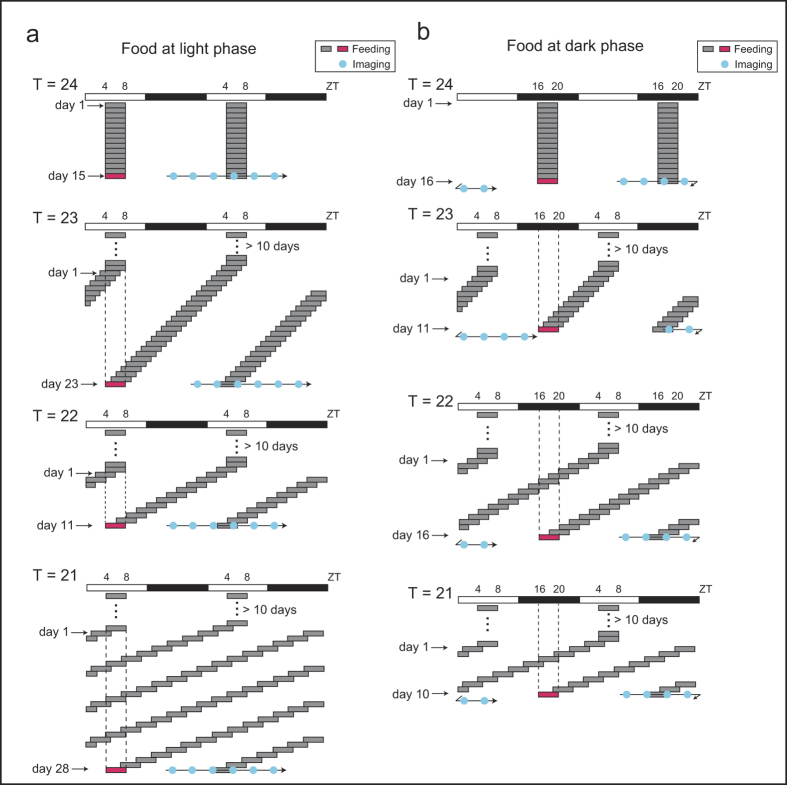
Experiment 1 schedule. In experiment 1, each T-cycle experiment of scheduled feeding was performed under normal light-dark cycles after adaptation to a Tau (T) = 24-h cycle of food intake at zeitgeber time (ZT; ZT0 is defined as the time when the light was switched on) 4–8 for a minimum of 10 days. Each T-cycle of scheduled feeding was then maintained with specific food timings for a minimum of 10 days (detailed number of days are indicated on the left). The PER2::LUCIFERASE (PER2::LUC) bioluminescence rhythm (the six measurement time points per day are indicated by arrows with blue circles) was monitored after food intake (red boxes) at ZT4–8 (light phase, **a**) or ZT16–20 (dark phase, **b**). Horizontal open and closed bars indicate light and dark periods, respectively.

**Figure 2:**
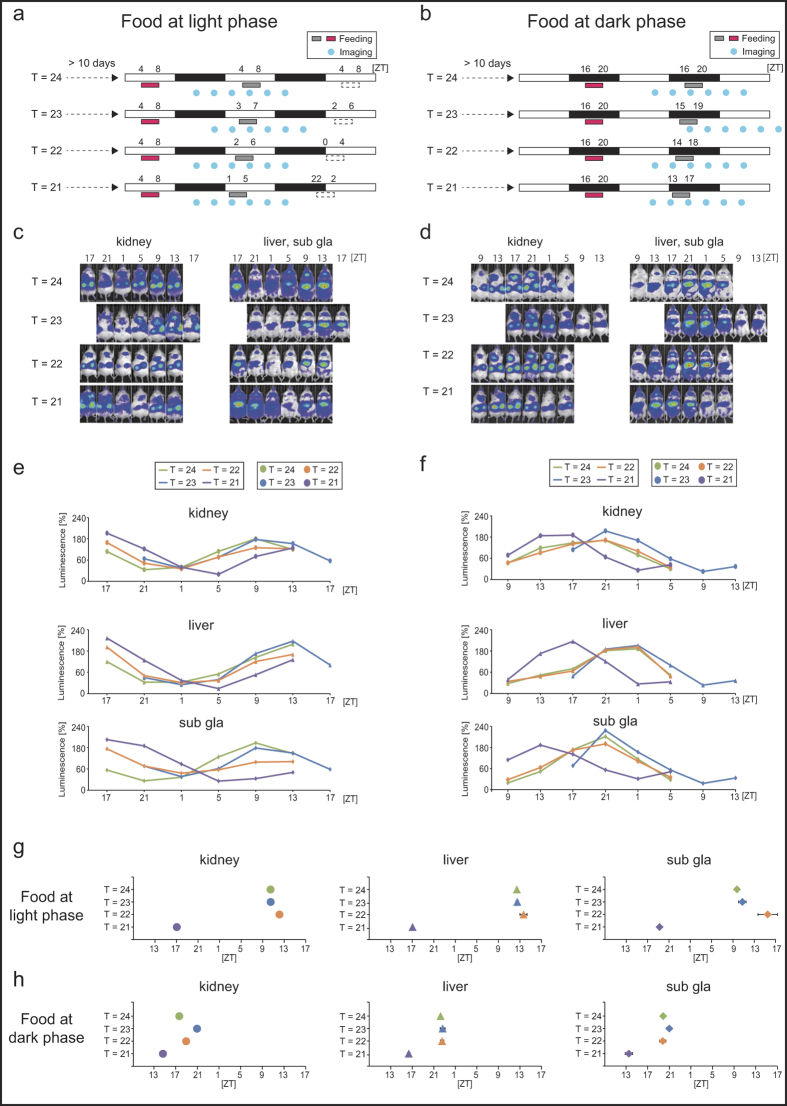
Peripheral PER2::LUCIFERASE (PER2::LUC) bioluminescence under Tau (T) = 21–24-h conditions (Experiment 1). (**a**,**b**) Summary of experiment 1 schedules (as in Fig. 1). Each T-cycle of scheduled feeding (4 h of feeding time, at an interval of 21–24 h) was maintained for at least 10 days, and the PER2::LUC bioluminescence rhythm (the six measurement time points per day are indicated by blue circles) was monitored after food intake (indicated as red boxes) at zeitgeber time (ZT; ZT0 is defined as the time when the light was switched on) 4–8 (light phase, **a**,**c**,**e**,**g**) or ZT16–20 (dark phase, **b**,**d**,**f**,**h**). Horizontal open and closed bars indicate light and dark periods, respectively. (**c**,**d**) Representative images of the PER2::LUC rhythm in the kidneys, liver, and submandibular gland (Sub gla) under each T-cycle. In the T = 23-h group, the recording time schedule was different from that of the other groups because of some issues with the recording apparatus. (**e**,**f**) Average waveforms of the PER2::LUC rhythm. (**g**,**h**) Calculated peak phases of the PER2::LUC rhythm. Data are presented as mean ± s.e.m. The number of mice used in this study is listed in Table S1.

As shown in Figs 1 and 2a,b, each T-cycle experiment of scheduled feeding was performed under normal light-dark cycles after adaptation to a T = 24-h cycle of food intake at zeitgeber time (ZT; ZT0 is defined as the time when the light was switched on and ZT12 is defined as the time of light off) 4–8 for a minimum of 10 days. The mice were then fed under specific T-cycle conditions for at least 10 days, and PER2::LUCIFERASE (PER2::LUC) bioluminescence rhythms (six imaging time points per day) in the kidneys, liver, and submandibular gland were measured. We previously reported that this protocol (anesthesia) did not affect the evaluation of the molecular clock rhythm in peripheral organs^16^. We divided the mice into two groups (Fig. 1a vs. 1b; Fig. 2a vs. 2b) and measured the bioluminescence rhythm after food intake at ZT4–8 (light phase) or ZT16–20 (dark phase) under each T-cycle. We would expect the anti-phase of the PER2::LUC rhythms to be present between these two groups if the peripheral clock is entrained by the T-cycle condition, and if the clock is not entrained, we would expect to the cycle to be in the same phase (see Fig. 2g,h). In preliminary experiments, we examined the phase of the peripheral clock 10 days after switching from night time feeding (ZT16–20) to daytime feeding (ZT4–8) under normal light-dark cycles. The clock phases of the organs of these animals (kidneys: ZT8.8 ± 0.5, liver: ZT11.6 ± 0.1, submandibular gland: ZT9.9 ± 0.2) were identical to those of mice kept for 15 days with a T = 24-h cycle during the light period (Figs 1a and 2g), suggesting that 10 days is a sufficient time period to change the phase of peripheral clocks to the anti-phase by scheduled feeding. Body weight decreased in all T-cycle-condition groups compared to that in the T = 24-h group, but this decrease in each group was only <10% (Fig. S1a and S1b). Similar changes were observed in food intake among the T = 24–21-h groups (Fig. S1c and S1d). Fitting analysis was performed to the cosine curve for the waveforms of the bioluminescence rhythm. Arrhythmic or low amplitude oscillations were eliminated from the analysis (Table S1 and Fig. S2). In the current study, most tissues in the experimental groups showed rhythmic and normal amplitude of PER2::LUC oscillations. The peak phases in the tissues of the T = 22–24-h cycle group showed an anti-phasic behaviour between the two different measurement schedules (at food intake in the light phase and food intake in the dark phase) (Fig. 2g,h), suggesting that PER2::LUC oscillations in each tissue adapt to the specific feeding T-cycle and run within that specific period. However, under the T = 21-h schedule, the peak phases in each tissue showed similar ZT points for the two measurement schedules (Fig. 2g,h), suggesting that they could not be entrained to the T = 21-h cycle.

### Experiment 2: Effects of scheduled feeding with T = 15, 18, and 21-h cycles on the period of the peripheral clock

**Figure 3:**
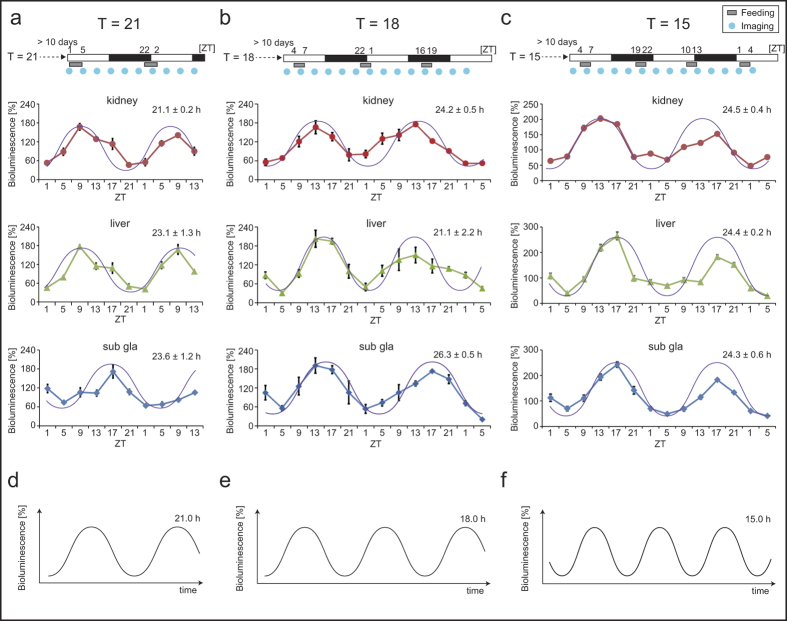
Peripheral PER2::LUCIFERASE (PER2::LUC) bioluminescence under Tau (T) = 15–21-h conditions (Experiment 2). Experimental schedules (upper). After adaptation to a T = 24-h cycle of food intake at zeitgeber time (ZT; ZT0 is defined as the time when the light was switched on) 4–8 for a minimum of 10 days, each T-cycle of scheduled feeding (3 h of feeding time for T = 15 and 18 h; 4 h of feeding time for T = 21 h) was maintained for a minimum of 10 days, and the PER2::LUC bioluminescence rhythm was monitored at the indicated time points (blue circles). Bioluminescence imaging was performed at 10 time points during 40 h for T = 21 h, or 14 time points during 56 h for T = 18 and 15 h. The averaged waveform of PER2::LUC bioluminescence under T = 21 (**a**, n = 4), 18 (**b**, n = 3), and 15 h (**c**, n = 8) of T-cycle of scheduled feeding (lower). Data are presented as mean ± s.e.m. Fitting analysis to the cosine curve was performed, and the results for the fitting period are presented in each panel. The purple line indicates the cosine curve, with each calculated period fitted to bioluminescence data. (**d–f**) Ideal examples of the cosine curve for every period of each feeding cycle (T = 21, 18, and 15 h). The time scale presented in the x-axis (**d–f**) is the same as that shown for each T-cycle data set (**a–c**).

If PER2::LUC oscillations in the peripheral clock were entrained to the T = 15–21-h feeding schedule, waveforms should show oscillations with a shorter period (≤21 h). Therefore, we continuously monitored bioluminescence rhythms for a couple of days under T = 15, 18, and 21-h conditions. In fact, bioluminescence imaging was performed at 10 consecutive time points during 40-h for T = 21 h, or 14 time points during 56-h for T = 18 and 15 h (Fig. 3). The food intake decreased in the T = 18 and 15-h groups from that of the T = 24-h group because the duration of feeding time was shorter for the former two groups (3 h) than for the T = 24-h group (4 h) (Fig. S1c). Additionally, in the T = 18 and 15-h groups, the body weight showed a similar decrease from that observed in the T = 21–23-h groups (Fig. S1a). The fitting analysis to the cosine curve revealed an oscillation period (21.1–26.3 h) in each tissue (Fig. 3). The same period between the feeding and peripheral clock cycles was found in the kidneys of the T = 21-h (21.1 ± 0.2 h) animals, but the corresponding periods in the liver and submandibular gland were not fitted to the cosine courve in the T = 21-h (23.1 ± 1.3 h for the liver; 23.6 ± 1.2 h for the submandibular gland), suggesting that T = 21 h is out of the range of food entrainment in the liver and submandibular gland. In addition, under the T = 15 or 18-h conditions, each fitting period was not fitted to the feeding cycle. The PER2::LUC oscillations in each tissue (Fig. 3d–f) differed from the cosine curve (T = 15 or 18 h). Thus, our data suggest that the peripheral clock could not be entrained by the T = 15 or 18-h feeding cycle.

## Discussion

In this study, we investigated the range of entrainment of peripheral clocks to shorter T-cycles (T ≤ 24 h) of scheduled feeding using the *in vivo* PER2::LUC monitoring method. We found that peripheral clocks could be entrained to the T = 22–24-h cycle, but not to the T = 15–21-h cycle. In the case of T = 21 h, the PER2::LUC rhythm in the kidneys (Fig. 3) seemed to be entrained, unlike that in other tissues (Figs 2 and 3). In the case of T = 15 or 18 h, the PER2::LUC rhythm in each tissue showed daily oscillations with the ~24 h period and showed peak phases similar to those observed in the T = 24-h group with food intake during the dark phase (Fig. 2h). This indicates that peripheral clocks entrained by light-dark cycles appeared under these conditions. Thus, peripheral clocks could be entrained to a T-cycle with a 22–24-h period under light-dark cycles. This conclusion may be supported by our previous results, in which scheduled feeding in the middle of the day caused the mouse peripheral clock to advance by 2–3-h per day^19^; peripheral clocks have an ability to run with a 21–22-h period. On the other hand, Stokkan *et al.*^20^ reported that the speed of the phase shift of the rat liver clocks by 4-h feeding in the daytime was 5–10-h per day, which is not consistent with our current results. While masking responses to the schedule feeding could explain this rapid and acute phase shift, one could also conclude that the rat liver clock has the capacity to respond to the scheduled feeding with a shift of 5–10-h per day. This would place the limits of liver entrainment well outside the 21–27-h window set by the SCN and is in direct contrast with our suggestion of a 2–3-h shift per day^19^. Therefore, this should be addressed in future research and would be better supported by experiments in SCN-lesioned mice or in mice housed under light-light conditions.

In the present experiment, the peripheral clocks of the mice failed to entrain to the T <22 h scheduled feeding; this differed from the previously published data showing the entrainment of anticipatory activity to T = 12-h scheduled feeding^12^, suggesting that the mechanism of these oscillations was different. The possible explanation for this difference is that some oscillators for food anticipatory activity corresponded to one peak, while other oscillators corresponded to a second peak, when two peaks of anticipatory activity were observed during 12-h feeding cycles. In previous experiments, we and others observed peripheral bioluminescence oscillations with a 24-h period even with a feeding cycle set to the T = 12-h period^12^,^17^,^18^, which is consistent with the current results. Therefore, entrainment of peripheral clocks to the scheduled feeding was independent of the appearance of food anticipatory activity.

The experimental conditions affect the results for the range of T-cycle entrainment. Free access to running wheels has been shown to promote entrainment to the T-cycle of the light-dark cycle in mice^21^,^22^. Food anticipatory activity is reduced in the presence of light, as shown previously by the light-induced direct inhibition of this activity in nocturnal animals^23^. The SCN inhibits the formation of anticipatory activity rhythm. In fact, SCN lesions were shown to facilitate the formation of this rhythm^24^. In addition, animals with SCN lesions show a wider range of entrainment to scheduled feeding than sham-operated animals, as determined using food anticipatory activity^9^,^11^. Thus, there is an interaction between photic and feeding entrainment in the anticipatory activity rhythm and the entrainment of peripheral clocks^25^. If the T-cycle of feeding is examined under constant dark or constant light conditions, or by using mice with SCN lesions to reduce the SCN-dependent entrainment, it might become difficult to determine the phase of peripheral clocks when they are out of the T-cycle range. This is due to the fact that mice with SCN lesions showed dampened peripheral clock oscillations^16^. Therefore, we performed the present experiments using SCN-intact mice under normal light-dark conditions. The scheduled-feeding-induced shift in the peripheral clock phase (i.e. *Per2* in the liver and/or kidneys) is dependent on the trial days and food components of scheduled feeding and on meal size^17^,^18^,^19^. Therefore, the experimental protocol and schedule of the T-cycle may influence the range of entrainment in the present experiments. In several studies, the T-cycle experiment has been performed by gradually decreasing or increasing the period of stimulation (e.g. changing the period at a rate of 10 min/day) to promote entrainment^6^,^7^,^22^. However, in the present study, the mice were immediately exposed to the T-cycle from the T = 24-h conditions. In the current experiments, we examined scheduled feeding with T ≤ 24-h cycles, but not T > 24-h cycles, because long fasting periods followed by refeeding with T ≥ 24-h conditions may have other effects on the phase of peripheral clocks. In fact, longer fasting durations have a stronger resetting power for the peripheral clocks^17^,^18^. Additionally, the reduction of calorie intake affects the phase of the SCN clock as well as the peripheral clock^13^,^18^,^26^. Taken together, these data suggest that the experimental protocol influences the results in T-cycle experiments. In conclusion, we demonstrated a 22–24-h range of entrainment of mouse peripheral molecular clocks to shorter T-cycles of scheduled feeding in normal light-dark cycles, and this range is similar to that of T-cycles of light-dark stimulation.

## Methods

### Animals

All experimental protocols were carried out in accordance with the approved guidelines by the Committee for Animal Experimentation of the School of Science and Engineering at Waseda University (permission 2013-A061) and in accordance with the law (No. 105) passed by and the notification (No. 6) released by the Japanese government. Male, heterozygous PER2::LUC knock-in mice^27^ (age: 3–6 months; provided courtesy of Dr Joseph Takahashi, University of Texas Southwestern Medical Center, TX, USA) were used in this study. PER2::LUC knock-in mice (C57/BL6) were backcrossed (6–7 times) with imprinting control region (ICR) (albino) mice to monitor bioluminescence by *in vivo* imaging. Mice were maintained under a light-dark cycle (12-h light and 12-h darkness, with lights being switched on at 8:00 a.m.), at a room temperature of 23 °C ± 1 °C, humidity of 60% ± 5%, and light intensity of 100–150 lux at the cage level. They were supplied a standard diet (MF; Oriental Yeast Co. Ltd., Tokyo, Japan) and *ad libitum* access to water before initiation of the experiments.

### *In vivo* monitoring protocol

*In vivo* monitoring was performed as previously described^16^. *In vivo* imaging utilised an IVIS kinetics system (Caliper Life Sciences, MA, USA, and Summit Pharmaceuticals International Corporation, Tokyo, Japan). Mice were anaesthetised with isoflurane (Mylan Inc., Tokyo, Japan) and concentrated oxygen (SO-005B; Sanyo Electronic Industries Co. Ltd., Okayama, Japan) using a gas anaesthesia system (XGI-8; Caliper Life Sciences), inside a black box. While the mice were under anaesthesia, they were injected with d-luciferin potassium salt (Promega, WI, USA) subcutaneously in the back, near to the neck, and the dose was 15 mg/kg (30 mg/10 mL, 0.05 mL/10 g body weight). Images were captured 8 min after luciferin injection in the dorsal-up position for the kidney and 10 min after injection in the ventral-up position for the liver and submandibular gland. Images were captured using an exposure time of 1 min. For each time point, the bioluminescence image was merged with the grey-scale image. To investigate the circadian oscillation of PER2::LUC in peripheral tissues, images were obtained a minimum of six times per day at 4-h intervals. This manipulation had no effect on the PER2::LUC rhythms^16^. The average photon/s value of the data from each day was designated as 100%, and the bioluminescence rhythm for the entire day was expressed as a percentage of each set for the individual organs. The peak phase and amplitude of this normalised percentage data were determined using the single cosinor procedure with a 24-h period for the data in Fig. 2 (acro.exe; designed by Dr Refinetti^28^). Data with low amplitude (<40%) or high goodness of fit value (>0.1) were defined as “arrhythmic” and eliminated from data analysis. The oscillation period was determined using the cosinor procedure with a wide range of periods (15–27 h) for the data in Fig. 3 (Cosinor.exe; designed by Dr Refinetti^28^).

### Scheduled feeding protocol

The scheduled feeding protocol, in which mice had free access to food during the feeding period, was conducted using an automatic feeding apparatus as previously described^29^. The feeding period for food was 4 h in the T = 24, 23, 22, and 21-h groups and 3 h in the T = 18 and 15-h groups because the interval between meal times became shorter in the latter two groups.

### Statistical analysis

Data were analysed using GraphPad Prism (version 6.03; GraphPad software, CA, USA). Equal variance and normal distribution tests were performed to select the appropriate statistical approach. Parametric analyses were conducted using a one-way analysis of variance with Dunnett’s test for post hoc analysis, and non-parametric analysis was carried out using a Kruskal-Wallis test with Dunn’s test for post hoc analysis. Data are expressed as the mean + or ± s.e.m. P < 0.05 was considered statistically significant.

## Additional Information

**How to cite this article**: Hamaguchi, Y. *et al.* Entrainment of mouse peripheral circadian clocks to <24 h feeding/fasting cycles under 24 h light/dark conditions. *Sci. Rep.* **5**, 14207; doi: 10.1038/srep14207 (2015).

## Supplementary Material

Supplementary Information
