# Supplementary material for: Entrainment of mouse peripheral circadian clocks to <24 h feeding/fasting cycles under 24 h light/dark conditions
Source: Sci Rep. 2015 Sep 23;5:14207. doi: 10.1038/srep14207 (PMC4585804; doi:10.1038/srep14207)
Supplement: Supplementary Information [file srep14207-s1.pdf]

Supplementary information

Entrainment of mouse peripheral circadian clocks to <24 h feeding/fasting cycles under 24 h light/dark conditions

Yutaro Hamaguchi[1, 2], Yu Tahara[1, 2], Hiroaki Kuroda[1], Atsushi Haraguchi[1], and Shigenobu Shibata[1*]

[1]Laboratory of Physiology and Pharmacology, School of Advanced Science and Engineering, Waseda University, Tokyo, Japan

[2]These authors contributed equally to this work.

[*]**Correspondence and proofs should be sent to:**

Shigenobu Shibata

Laboratory of Physiology and Pharmacology, School of Advanced Science and Engineering, Waseda University, Wakamatsu-cho 2-2, Shinjuku-ku, Tokyo 162-8480, Japan

Telephone: +81-3-5369-7318; Fax: +81-3-3341-9815; E-mail: shibatas@waseda.jp

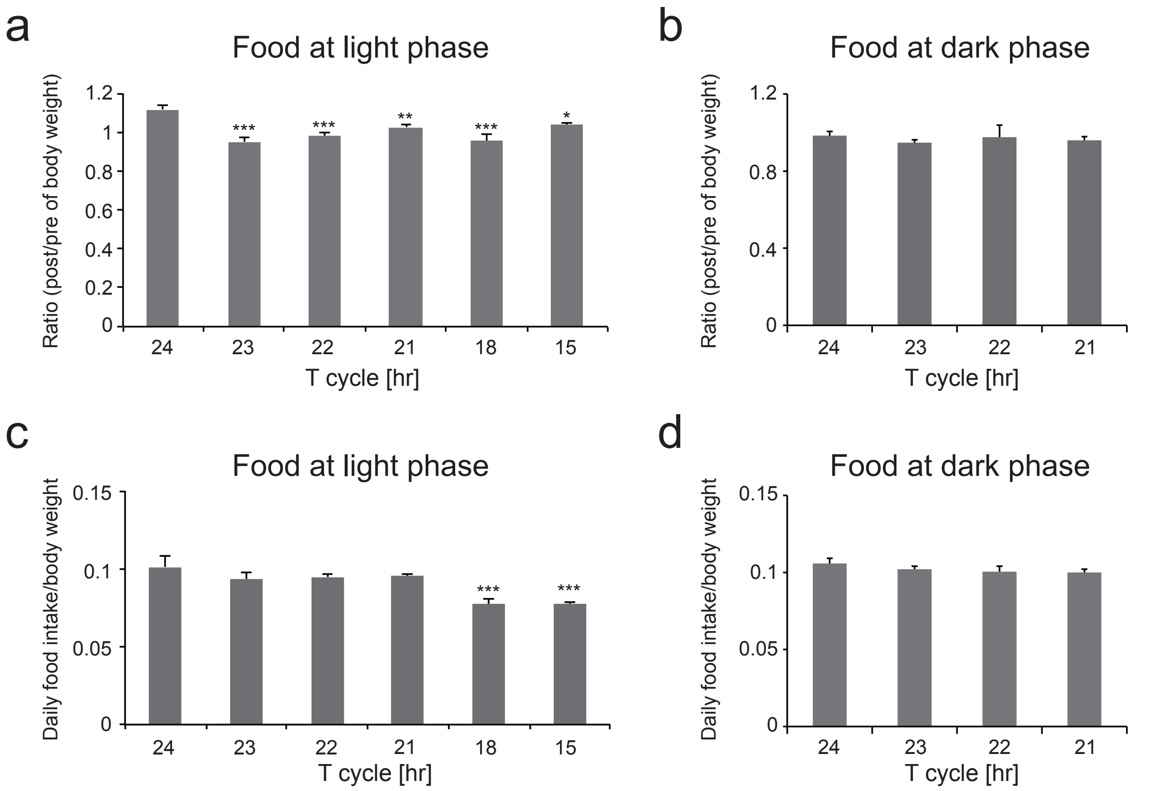

Figure S1. Body weight change and food intake in each experiment, related to Fig. 2 and 3

(**a, b**) Change in body weight after each T-cycle condition. Body weight was measured on the first day of each T-cycle (pre) and on the day of bioluminescence measurement (post). Body weight on the starting day was set as 1. (**c, d**) Daily food intake/body weight during each T-cycle condition. Data are shown as mean ± s.e.m. *P < 0.05, **P < 0.01, ***P < 0.001, vs. T = 24 h by Dunnett test.

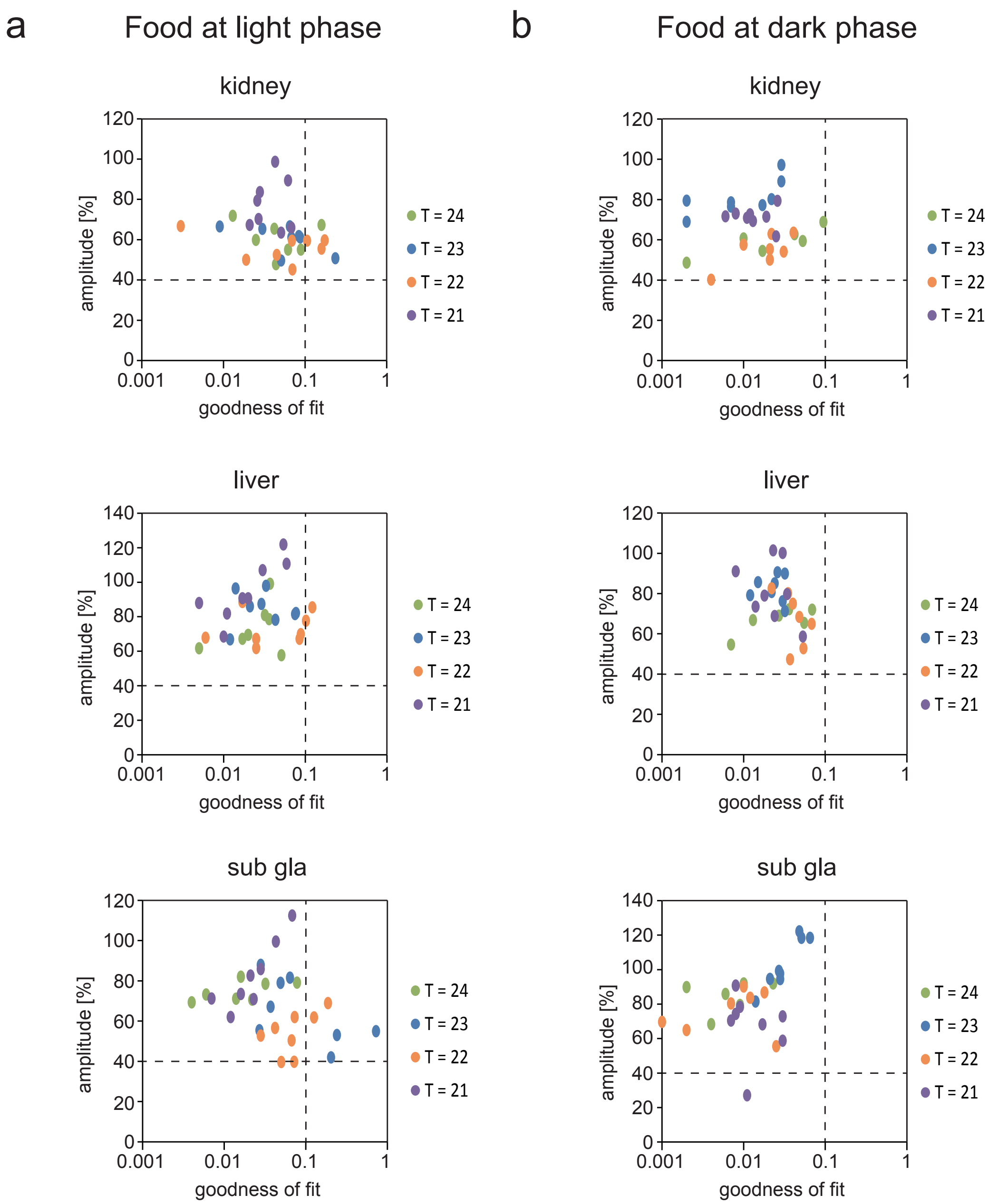

Figure S2. Correlation map of amplitude and goodness of fit value of PER2::LUC rhythms, related to Fig. 2

Correlation map of amplitude and goodness of fit in each tissue of T = 21–24 h condition with food intake during light phase (**a**) and dark phase (**b**) analysed by cosine curve fitting software (acro.exe). Each plot indicates the data of each mouse tissue. PER2::LUC rhythm with high amplitude (>40%) and low goodness of fit value (<0.1) was defined as “rhythmic” (Tahara et al., 2012).

Table S1. Number of mice examined and tissues that met (passed) rhythmicity criteria in each experimental group

T-cycle (hr)	feeding time	number of mice	number of tissue samples (pass/total)		
			kidney	kiver	sub gla
T = 24	light phase	7	6/7	7/7	7/7
	dark phase	6	6/6	6/6	6/6
T = 23	light phase	8	7/8	8/8	5/8
	dark phase	8	8/8	8/8	8/8
T = 22	light phase	8	5/8	6/8	4/8
	dark phase	7	7/7	7/7	7/7
T = 21	light phase	8	8/8	8/8	8/8
	dark phase	8	8/8	8/8	7/8

Cosinor analysis was performed for PER2::LUC bioluminescence. PER2::LUC rhythms with an amplitude of >40% and a goodness of fit value of <0.1 were defined as “rhythmic”.
